# ZnO nanoparticles induce acute arrhythmia and heart failure in mice by disturbing cardiac ion channels

**DOI:** 10.3389/fcvm.2025.1569265

**Published:** 2025-05-30

**Authors:** Xuexue Liu, Hongmei Deng, Zijuan Zhao, Shan Wen, Kailong Ma, Xinyu Wang, Yanfei Du, Chang Li, Jun Li, Guangqin Zhang, Xiaobo Zhou, Tuck Wah Soong, Ziqi Yuan, Jian Feng, Guang Li

**Affiliations:** ^1^ Department of Laboratory Medicine, Sichuan Province Engineering Technology Research Center of Molecular Diagnosis of Clinical Diseases, Molecular Diagnosis of Clinical Diseases Key Laboratory of Luzhou, The Affiliated Hospital of Southwest Medical University, Luzhou, China; ^2^ Department of Cardiology, the Affiliated Hospital of Southwest Medical University and Key Laboratory of Medical Electrophysiology, Ministry of Education & Medical Electrophysiological Key Laboratory of Sichuan Province, Institute of Cardiovascular Research, Southwest Medical University, Luzhou, China; ^3^ Department of Clinical Pharmacy, China Pharmaceutical University, Nanjing, China; ^4^ Department of Physiology, Yong Loo Lin School of Medicine, National University of Singapore, Singapore, Singapore

**Keywords:** zinc oxide nanoparticles, heart failure, cardiac arrhythmia, ion channels, calcium homeostasis

## Abstract

**Background:**

The widespread use of zinc oxide nanoparticles (ZnO NPs) has raised safety concerns on human health. However, the effects and underlying mechanisms of ZnO NPs exposure on the heart, especially during acute exposure, have yet to be elucidated.

**Methods:**

Two different sizes of ZnO NPs (40 nm and 100 nm) were selected and their in vivo effects on mouse heart were evaluated by echocardiography and electrocardiograms. Action potential, ion channel currents, and calcium recordings were employed to assess the electrical alterations in individual myocytes. The underlying mechanisms were further investigated by transmission electron microscopy (TEM) imaging, mitochondrial staining, LDH and ROS detection. In addition, human-induced pluripotent stem cell-derived cardiomyocytes (hiPSC-CMs) were utilized for translational exploration.

**Results:**

Acute exposure to ZnO NPs induces cardiac dysfunction and arrhythmia in mice. Mechanistically, exposure to ZnO NPs did not significantly affect the IK1, but it markedly decreased I_Na_ and I_Ca−L_ currents, resulting in a reduced amplitude and shortened duration of the action potential in cardiomyocytes. These changes not only prolonged PR-interval and blocked A-V conduction that triggered cardiac arrhythmia, but also led to a diminished calcium transient, which contributed to heart failure. The downregulation of calcium transient upon ZnO NPs exposure was further confirmed in hiPSC-CMs. Meanwhile, acute exposure to ZnO NPs did not induce endocytosis, impair membrane integrity, or promote ROS production in the mitochondria of cardiomyocytes.

**Conclusion:**

Acute ZnO NPs exposure causes heart failure and arrhythmia in mice by directly impacting ion channel function.

## 1 Introduction

Zinc oxide nanoparticles (ZnO NPs) are widely utilized in various fields closely related to daily lives, such as cosmetics, food industry, textiles, and biomedicine (1). The extensive usages of ZnO NPs can be attributed to their exceptional absorption capabilities, excellent biocompatibility, antibacterial activity, and antioxidant properties. They can serve as nutritional supplements, antibacterial agents, and contribute to food storage, packaging, and quality control (2, 3). In biomedical field, ZnO NPs are supposed to have potential applications in anticancer, antibacterial, anti-inflammatory, drug delivery, and biological imaging (4, 5). However, the widespread usages of ZnO NPs have also raised concerns about their biosafety. Synthetic ZnO NPs can enter the human body through skin, respiratory tract and digestive tract, as well as through biomedical exposure, such as drug transporters (6–9). It has been reported that ZnO NPs promote the generation of reactive oxygen species (ROS) that leads to cytotoxicity in different cell types and organs of mammalian (10–12).

The harmful effects of ZnO NPs on the cardiovascular system have also been noticed since ZnO NPs can be deposited in the heart upon exposure (13). *In vitro*, Poier et al. reported that prolonged exposure to ZnO-NPs (average size: 45–55 nm) induce DNA damage of HUVEC at concentrations exceeding 25 μg/ml, indicating their potential toxicity in angiogenesis (12). Yuka et al. reported that 16 h exposure to ZnO NPs, with a primary diameter of 20 nm, promote migration and adhesion of THP-1 monocytes to HUVECs, suggesting that they promote foam cell formatting during atherosclerogenesis (14). The cytotoxicity of ZnO NPs on excitable cardiomyocytes was also reported. ZnO nanowire arrays could inhibit the metabolism of HL-1 cardiac muscle cell lines and primary neonatal rat cardiomyocytes (15), whereas ZnO NPs with around 100 nm size analyzed by TEM inhibited the proliferation of H9c2 rat cardiomyoblasts, and induced apoptosis and necrotic cell death after 48 h of treatment (16). *In vivo*, Manigandan et al. demonstrated that ZnO-NPs with particle sizes <100 nm affects cardiovascular development of chicken embryos via elevated oxidative stress (17). Wang et al. further revealed that ZnO-NPs with particle sizes around 50 nm disturb heart tube formation of chicken/mouse embryo models in a dose-dependent manner (18). In adult male Wistar rats, ZnO-NPs with particle sizes around 39 nm could induce cardiac electrophysiological dysfunction and structural lesions after exposure via i.p. injections (100 mg/kg body weight) for up to ten days (19). Moreover, ZnO NPs with the particle size ≤50 nm, promoter ROS generation and perturbed mitochondrial biogenesis that led to electric dysfunction in human-inducible pluripotent stem cell-derived cardiomyocytes (iPS-CMs) (20). These observations indicated that ZnO NPs exposure not only damages cardiovascular development, but also affects cardiac function.

Cardiac function is tightly correlated with its electrical stimulation and mechanical contraction in a well-coordinated manner named as excitation-contraction (EC) coupling (21). Ion channels play a central role in EC coupling by maintaining cardiac excitability, conductivity, calcium homeostasis and contractility of cardiomyocytes. Ions flowing through ion channels initiates dynamic changes of transmembrane potential (TMP), which encompasses the resting potential (RP) and action potential (AP), and represents the potential difference between the inner and outer membranes of cardiomyocytes. In ventricular myocytes, the inwardly rectifying potassium current (I_K1_) primarily contributes to the resting potential, whereas other potassium currents including delayed rectifier potassium current (I_K_), together with depolarizing voltage-gated sodium current (I_Na_) and long-lasting calcium current (I_ca−L_), determine the shape and duration of AP (22). Influx of I_ca−L_ during AP triggers Ca^2+^ release from the sarcoplasmic reticulum (SR), which in turn initiates contraction of myocytes. Therefore, pathological disturbance of ion currents not only changes the AP waveform and affect cardiac excitation but also affects Ca^2+^ homeostasis and cardiac contraction (23).

Currently, the effect and underlying mechanism of ZnO NPs exposure on cardiac function, especially during acute exposure, is still unknown. In this study, two different sizes of ZnO NPs (40 nm and 100 nm) were selected to investigate their cardiovascular effects upon acute exposure. The *in vivo* significance was assessed by surface electrocardiogram (ECG), high-resolution 2D echocardiography system, and wireless telemetry system for awake mice. In addition, patch clamp recording was performed to test changes of AP and ion channel currents of myocytes, and calcium transients of both mouse cardiomyocytes and human iPS-CMs were recorded to investigate the alteration of E-C coupling upon ZnO NPs exposure. The involvement of oxidative stress and mitochondrial damages were also evaluated in a time-dependent manner. This study provides a new insight into the biosafety assessment of ZnO NPs on cardiovascular system.

## 2 Materials and methods

### 2.1 Preparation and characterization of ZnO NPs

ZnO NPs with sizes of 40 nm and 100 nm (ZnO NPs-40 and ZnO NPs-100, respectively) were purchased from Sigma (>97%, St. Louis, MO, USA). Before the experiments, the ZnO NPs stock solution was dispersed by sonication and vortex oscillation. ZnO NPs working solution were prepared and diluted freshly before each experiment. For cellular experiments, basal culture medium or corresponding bath solutions were used as dilute solution, whereas physiological saline was selected for animal experiments.

The morphology of ZnO NPs was observed by transmission electron microscope (HT7800, HITACHI, Japan). ZnO NPs were diluted with DMEM, dropped onto a copper grid, stained with 2% phosphotungstic acid, and observed under the TEM.

The physicochemical properties of ZnO NPs suspensions were determined using dynamic light scattering (DLS). Briefly, ZnO NPs-40 and ZnO NPs-100 (both 10^−3^ g/ml) were dispersed in normal saline, basal medium, or corresponding bath solutions, mixed well, and incubated for 5 min. The Nano ZS analyzer (Malvern Instruments Ltd, Malvern, UK) measured ZnO NPs' hydrodynamic size and zeta potential in solution. Measurement of Zn^2+^ concentration released by ZnO NPs in storage solution using inductively coupled plasma emission spectroscopy (ICP-OES, Avio 500, PerkinElmer). ZnSO_4_·7H_2_O (1 M) was used as a positive control. Detailed schematic diagram illustrating the experimental design, key procedures, and critical time points for assessments across each model can be found in Supplementary Figure S1.

### 2.2 Echocardiography

All mice underwent echocardiographic measurements using a Vevo 3100 system (FUJIFILM Visual Sonics, Inc.). 8–10 weeks male C57BL/6J mice were anesthetized with isoflurane and echocardiograms were recorded before exposure to ZnO NPs (baseline) and continuously recorded after exposure for 10 min. Only one injection was placed in each mouse for 100 μl solution. The ZnO NPs were administered intravenously (i.v.) at doses of 10 mg/kg body weight (bw) or 20 mg/kg bw, or 100 μl of normal saline was administered alone to the control animals. To investigate the direct impact of ZnO NPs on cardiac function, echocardiographic parameters including heart rate, left ventricular ejection fraction (LVEF), and left ventricular fractional shortening (LVFS) were calculated before and after ZnO NPs exposure.

### 2.3 Surface electrocardiogram

To examine the possible arrhythmogenic consequences of ZnO NPs *in vivo*, we conducted surface electrocardiograms (ECGs) to record and evaluate any potential effects. 8–10 weeks male C57BL/6J mice were anesthetized with isoflurane, and ECGs were collected using a BIOPAC MP150 ECG data acquisition module (BIOPAC Systems, Inc., Goleta, CA, USA) before and after exposure to ZnO NPs for up to 60 min. ZnO NPs-40 were intravenously injected at doses of 5, 8, 10, and 20 mg/kg, while ZnO NPs-100 were administered at doses of 5, 10, 20, and 30 mg/kg. Both types of ZnO NPs were diluted to the desired concentrations in normal saline. Each mouse received an injection of 100 μl. Arrhythmia was continuously monitored for 60 min after injection. The BIOPAC data analysis package in AcqKnowledge 5.0 was used to extract quantitative metrics from the ECG signal.

### 2.4 Isolation and primary culture of neonatal mouse ventricular myocytes (NMVMs)

Neonatal mouse ventricular myocytes (NMVMs) were isolated from the ventricles of neonatal C57BL6/J mice (24–72 h after birth) by using trypsin and collagenase. Briefly, mice were sacrificed, and heart tissue was quickly dissected to separate the ventricles, then washed twice with pre-cooled PBS. Ventricular tissues were digested with 0.25% trypsin at 4°C overnight, followed by digestion with type II collagenase for 1 min at 37°C. Then terminate digestion with DMEM medium containing fetal bovine serum (FBS) and collect liquid, the steps were repeated several times. Cells were harvested by centrifugation. The NMVMs were carefully suspended again in DMEM. Cells were cultured at 37°C in a 5% CO_2_ atmosphere. After culturing for 24 h, cardiomyocytes were used for whole-cell patch-clamp experiments.

### 2.5 Cardiomyocyte differentiation from hiPSCs

The hiPSC line (HNF-P30-P11) was obtained from OSINGLAY, China, and they were differentiated into cardiomyocytes by using small molecule GSK3 and Wnt inhibitors as previously reported (24). In brief, hiPSCs were dissociated into single-cell suspensions using Versene solution (15040066, Gibco, USA) and resuspended in hiPSC maintenance medium, cultured until the colonies reached 80–90% confluence, and then began to differentiate. On day 0, the medium was changed to RPMI medium with B-27 supplement minus insulin, containing 5 μM CHIR-99021, an agonist of Wnt/*β*-catenin signaling pathway, and incubated for 48 h at 37°C in 5% CO_2_. Then the medium was replaced with RPMI/B-27 without insulin for another 24 h. From day 3 to day 5, the cells were subjected to a treatment of 5 μM IWP2 in RPMI/B-27 without insulin. On day 5, the medium was substituted with RPMI/B-27 without insulin, and the cells were further incubated for 48 h at 37°C in a 5% CO_2_ atmosphere. Cells were cultured in RPMI/B-27 with insulin medium from day 7 onwards. Contracting cells were observed from days 7–8 and thereafter. Subsequently, the cells were maintained in cardiomyocyte maintenance medium, which was replaced every 3 days.

### 2.6 Patch-clamp

The PatchMaster software (Heka Elektronik, Lambrecht, Germany) and EPC-10 patch-clamp amplifier were used with whole-cell patch-clamp technique to record TMPs and channel currents of cardiomyocytes. Glass microelectrode pipettes (resistance 3–5 MΩ) were pulled with a P-97 horizontal puller (Sutter Instruments, Novato, CA, USA) using standard wall borosilicate glass. KCl agar bridge were used and the junction potential were measured before and after application of ZnO NPs in bath solution.

The TMPs, including the RP and AP, were recorded using the current clamp mode. The bath solution contained (in mM): NaCl 137, KCl 5.4, MgCl_2_ 1, NaH_2_PO_4_ 0.33, Glucose 10, HEPES 10, and CaCl_2_ 1.8, and the pH value of the bath solution was adjusted to 7.4. The pipette solution contained (in mM): NaCl 9, potassium gluconate 123, phosphocreatine 14, HEPES 9, Mg-ATP 4, MgCl_2_ 1.8, and EGTA 0.9, and the pH value of the pipette solution was adjusted to 7.2. APs were elicited by rectangular current pulses of 1 Hz and 2 ms duration, with 200 pA as the minimum stimulation, stepping to 1,000 pA, and finally recording the AP with 1 or 1.5 X the minimum stimulation. The TMPs of cardiomyocytes were recorded and compared to baseline within 30 min of exposure to ZnO NPs-40 and ZnO NPs-100 (10^−6^–10^−4^ g/ml).

Voltage-clamp experiments were performed to record ion channel currents of cardiomyocytes pre- and post-exposure to various ZnO NPs concentrations for 5–30 min.

To record I_K1_, the bath solution contained (mM): NaCl 135, MgCl_2_ 1, KCl 5.4, HEPES 10, glucose 10, CaCl_2_ 1.8, and NaH_2_PO_4_ 0.33, and the pH of the solution was adjusted to 7.4 using NaOH. L-Ca channels were blocked using 10 μM nifedipine. The pipettes were filled with a solution containing mM of KCl 140, MgCl_2_ 1, HEPES 5, EGTA 10, K_2_-ATP 2, and 4-aminopyridine 5, and the pH was adjusted to 7.2 with KOH. I_K1_ was elicited by 500 ms depolarizing pulses stepping from −120 mV to +20 mV with 10 mV increments, starting from a holding potential of −20 mV.

To record I_Na_ and establish the I–V and activation curves of I_Na_ channels, the bath solution contained (in mM): NaCl 30, CaCl_2_ 1, MgCl_2_ 1, CsCl 105, nifedipine 0.01, glucose 5, HEPES 5, and was adjusted to pH 7.4. The pipette solution contained (in mM): CsCl 120, CaCl_2_ 1, MgCl_2_ 2, Na_2_ATP 2, HEPES 10, TEA 10, EGTA 11, and the pH was adjusted to 7.4 with CsOH. The I-V curves and activation were established using previously reported protocols (25, 26).

The bath solution that recorded I_Ca−L_ contained (in mM): NaCl 135, CsCl 4, MgSO_4_ 1.2, CaCl_2_ 1.8, HEPES 10, Glusoce 10, and the pH was adjusted to 7.4 with NaOH. The pipette solution contained (in mM): CsCl 120, MgCl_2_ 5, CaCl_2_ 1, HEPES 10, EGTA 10, Na_2_-ATP 5, and TAE 10, and the pH was adjusted to 7.25 with CsOH. The patch-clamp voltage was clamped at −50 mV for 50 ms, stimulated with a pulse width of 500 ms, with 10 mV steps, and a voltage stimulation from −50 mV–+50 mV was applied, with a sampling frequency of 10 kHz.

### 2.7 Calcium transients of cardiomyocyte

Dissociated neonatal mouse ventricular myocytes were plated and maintained on a 20 mm confocal glass-bottom culture dish for 48–72 h prior to the experiments. On the day of the experiment, the cells were incubated for 8 min with 5 μg/ml Fluo-4 AM Ca^2+^ indicator (Solarbio Life Science) in DMEM at 37°C. All recordings were performed in Tyrode's solution, which contained (in mM): NaCl 135, MgCl_2_ 1, KCl 5.4, glucose 10, NaH_2_PO_4_ 0.33, HEPES 10, taurine 5, CaCl_2_ 1.8, and pH adjusted to 7.4 with NaOH. Confocal microscopy was performed using a ZEISS LSM 980 inverted confocal microscope (Oberkochen, Germany). Fluo-4 was excited at 488 nm with an argon laser and the resulting emission intensity was measured at 510 nm. Caution was taken to avoid passing by the nucleus when scanning images of cardiomyocytes with a 512-pixel line positioned along the cell's longitudinal axis. Images were acquired in the X-Y mode. All images were taken using a Plan-Apochromat 40X (1.2 NA) objective (Zeiss). Line-scan kymographs were obtained for analysis. Ca^2+^ levels are presented as F/F0, where F0 is the minimum fluorescence intensity measured between contractions during the diastolic phase of the transient phase.

### 2.8 Lactate dehydrogenase assay *in vitro*

LDH levels in neonatal ventricular myocyte culture medium were measured using an LDH assay kit (Beyotime Biotechnology, Shanghai, China). Cardiomyocytes were seeded into 96 well plates, and after adhesion for 24 h, they were exposed to ZnO NPs-40 or ZnO NPs-100 at concentrations of 10^−3^–10^−6^ g/ml for 5–30 min. This experiment was conducted following the manufacturer's instructions. Absorbance was measured at 490 nm using an automatic microplate reader (Infinite 2000, TECAN, Switzerland).

### 2.9 Detection of mitochondrial ROS

Isolated neonatal cardiomyocytes were seeded into a 20 mm glass-bottom culture dish (Nest) for 48 h and then incubated with ZnO NPs-40 or ZnO NPs-100 at concentrations of 10^−6^–10^−4^ g/ml for 5–10 min. Controls were established using cells that were not treated with ZnO NPs. Cells were then incubated with MitoSOX Red working solution (Invitrogen, Carlsbad, CA, USA) and nuclei were stained with Hoechst 33342 (Beyotime Biotechnology, Shanghai, China). The fluorescence of MitoSOX Red (excitation, 510 nm) and Hoechst 33342 (excitation, 350 nm) was detected using a Carl Zeiss LSM 980 confocal microscope.

### 2.10 Mitochondrial staining

Isolated neonatal cardiomyocytes were seeded on a 20 mm glass-bottom culture dish (Nest) 48 h before treatment. The cells were incubated with ZnO NPs-40 or ZnO NPs-100 at concentrations of 10^−6^ g/ml and 10^−4^ g/ml for 10 min at 37°C and washed with PBS, then incubated with 100 nM MitoTracker Green (Beyotime Biotechnology) working solution at 37°C for 15 min. Nuclei were stained with Hoechst 33342. The fluorescence of MitoTracker Green (excitation, 490 nm) and Hoechst 33342 were detected using a Carl Zeiss LSM 980 confocal microscope.

### 2.11 Transmission electron microscopy (TEM) imaging

TEM was used to examine endocytosis of ZnO NPs and the organelle morphology of ventricular myocytes. Cells were fixed with 2.5% glutaraldehyde in 0.1 M PBS for 5 min, and then collected and centrifuged. Cell pellets were pre-embedded in agarose, post-fixed with 1% OsO_4_, dehydrated and embedded in epoxy resin. Ultrathin sections were cut with an ultramicrotome, mounted on copper grids, stained with a 2% uranium acetate saturated alcohol solution and 2.6% lead citrate. TEM images were obtained using a Hitachi HT7800 TEM.

### 2.12 Statistical analysis

Statistical analyses were performed using GraphPad Prism 8.4. Mean ± SEM is presented. Unpaired t-test or ANOVA with Bonferroni correction was used for comparisons between groups. *P* < 0.05 was considered statistically significant.

## 3 Results

### 3.1 Physicochemical characterization of ZnO NPs

The hydrodynamic diameters of ZnO NPs-40 (Supplementary Figure S2A) and ZnO NPs-100 (Supplementary Figure S2B) are consistent with a Gaussian distribution. The key physicochemical characteristics of the two ZnO NPs are summarized in Supplementary Table S1. The polydispersity index (PDI) was used to determine the uniformity of the particle size distribution of the nanoparticles. In this study, the zeta potentials of ZnO NPs-40 (Supplementary Figure S2C) and ZnO NPs-100 (Supplementary Figure S2D) in ultrapure water were lower than those in other solutions, and the PDI values were very low, indicating that the particle sizes of each system were relatively evenly distributed.

The concentrations of Zn^2+^ in the supernatants of ZnO NPs-40 and ZnO NPs-100 were 62.07 mg/kg (954.92 μM) and 36.47 mg/kg (561.08 μM), respectively. After diluted into working solution (10^−6^–10^−4^ g/ml), the concentration of Zn^2+^ from ZnO NPs is around 10^−6^–10^−4^ μM, which is far lower than physiological concentration (around 150 μM), indicating neglectable effects (27).

### 3.2 ZnO NPs cause complete A-V block in adult mice by prolonging PR-interval

**Figure 1:**
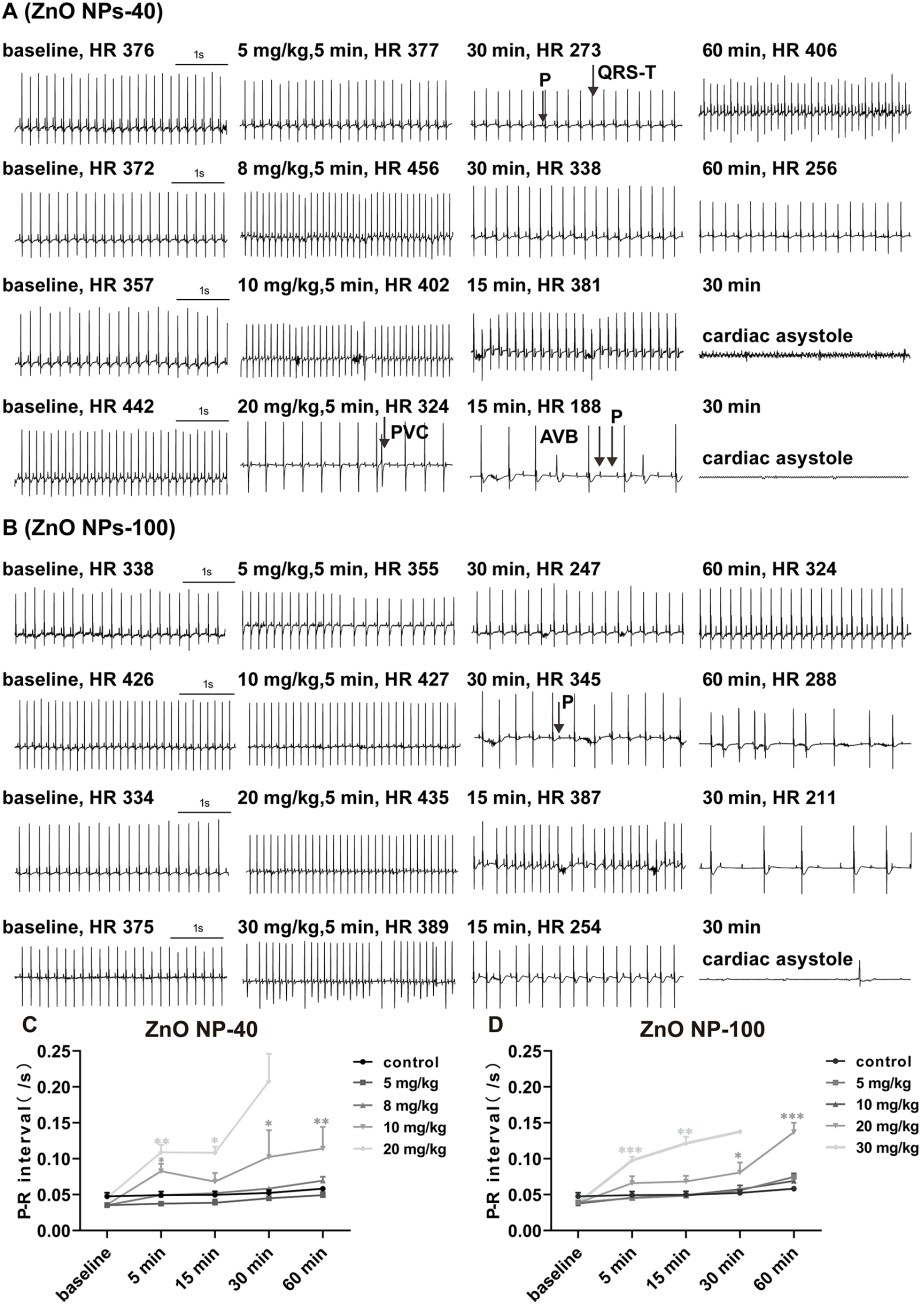
Effects of ZnO NPs on mice ECG. **(A)** Representative images showed the effects of ZnO NPs-40 at different doses and exposure duration on ECG in mice. **(B)** Representative images showed the effects of ZnO NPs-100 at different doses and exposure duration on ECG in mice. **(C)** Changes of PR-interval after different doses of ZnO NPs-40 for 0-60 min. **(D)** Changes of PR-interval after different doses of ZnO NPs-100 for 0-60 min. P, P wave; R, R wave; QRS-T, QRS complex and T wave; PVC, premature ventricular contractions; AVB, atrioventricular conduction block. *P < 0.05, **P < 0.01, ***P < 0.001. *n* = 6 for control group; *n* = 6 for ZnO NPs-40 at 5 mg/kg group and 8 mg/kg group; *n* = 11 for ZnO NPs-40 at 10 mg/kg group; *n* = 7 for ZnO NPs-40 at 20 mg/kg group; *n* = 6 for ZnO NPs-100 at 5 mg/kg group, 10 mg/kg group and 20 mg/kg group; *n* = 10 for ZnO NPs-100 at 30 mg/kg group.

To evaluate whether ZnO NPs have toxic effects on electrophysiology of C57BL/6J mice, we tested the effects of ZnO NPs-40 (Figure 1A) and ZnO NPs-100 (Figure 1B) of different doses on the surface ECG of mice. ZnO NPs-40 exposure at 5 mg/kg (i.v.) caused an initial increase followed by a gradual decrease of sinus heart rate (HR) over time, together with prolonged PR-interval. At a dosage of 10 mg/kg, ZnO NPs-40 not only caused similar changes of HR, but also induced premature ventricular contractions (PVC), significantly prolonged PR-interval, and led to complete atrioventricular conduction block (AVB) as early as in 5 min. Out of the eleven mice tested, seven died of cardiac asystole within 30 min. ZnO NPs-40 at 20 mg/kg rapidly caused PVC and AVB in 5 min, and gradually aggravated into complete AVB in 15 min; five of seven mice died of cardiac asystole within 30 min, and all of the tested mice died within the observed 60 min.

ZnO NPs-100 exposure at 10 mg/kg (i.v.) also increased HR initially, followed by a gradual slowing down of HR, prolonged PR-interval, PVC, and finally AVB within 60 min. ZnO NPs-100 at 20 mg/kg quickly prolonged the PR-interval in 5 min, followed by complete AVB, and cardiac asystole death (one of the six mice). ZnO NPs-100 at 30 mg/kg induced the same arrhythmia at a dose of 20 mg/kg; however, arrhythmia occurred earlier, and the incidence and mortality within the observed 60 min were higher than those in the 20 mg/kg group. As early as in 5 min, both ZnO NPs-40 and ZnO NPs-100 prolonged the PR-interval when the dozes were increased to 10 mg/kg and 20 mg/kg respectively (Figures 1C,D), indicating dose-, size-, and time-dependent effects of ZnO NPs exposure. After exposure to ZnO NPs-40 at a dose of 20 mg/kg or ZnO NPs-100 at 30 mg/kg for 30 min, only one or two mouse survived, which make it impossible for statistical analysis.

### 3.3 ZnO NPs inhibit depolarization and accelerate repolarization of myocardial action potential

**Figure 2:**
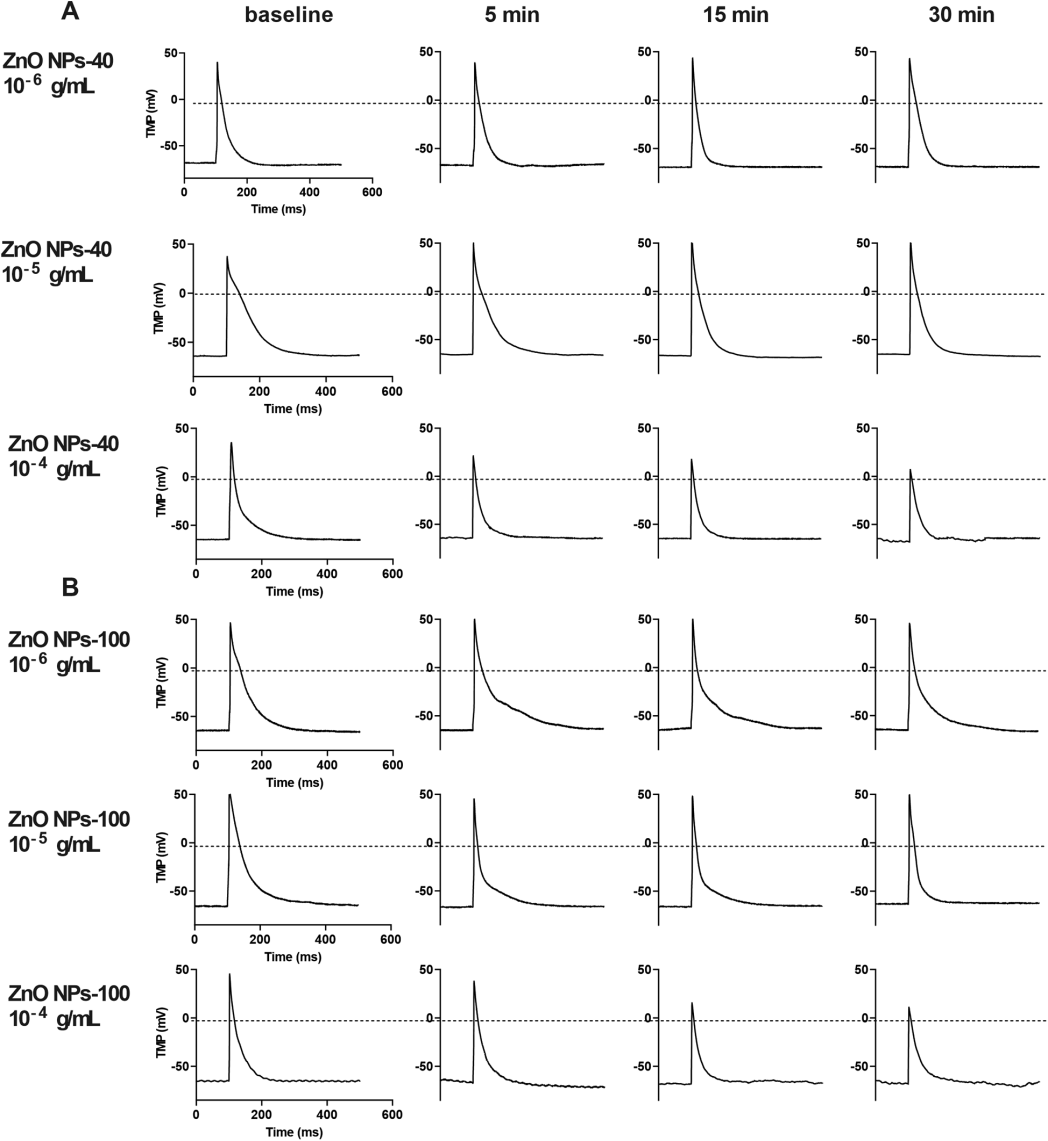
Representative image of transmembrane potential (TMP) of NMVMs before and after exposure to ZnO NPs-40 and ZnO NPs-100, respectively. **(A)** The effect of 10^−6^–10^−4^ g/ml ZnO NPs-40 on AP. **(B)** The effect of 10^−6^–10^−4^ g/ml ZnO NPs-100 on AP. AP. *n* = 10 for each group.

We then investigated the effect of ZnO NPs on cardiac electrophysiology of NMVMs by recording TMPs in current-clamp mode. At baseline, the RP of cardiomyocytes was −66.0 ± 7.5 mV, and the AP had a triangular shape without a typical plateau. The APA was 106.5 ± 12.9 mV and the time to 90% of action potential duration (APD_90_) was 94 ms. After exposure to ZnO NPs, even at the highest concentration (10^−4^ g/ml), both ZnO NPs-40 and ZnO NPs-100 had no significant effect on RP but significantly altered APA and APD as shown in Figures 2A,B.

ZnO NPs-40 at 10^−6^ g/ml had no significant effect on APA and APD, while exposure of ZnO NPs-40 at 10^−5^ g/ml significantly shortened APD_90_ after 30 min exposure although it had no significant effect on APA. ZnO NPs-40 at the highest test concentration (10^−4^ g/ml) significantly decreased the APA and APD as early as in 5 min, and further reduced APA (from 105.40 ± 3.61 mV–72.69 ± 8.42 mV, *P* < 0.05), the time to 10% action potential duration (APD_10,_ from 12.77 ± 0.28 ms–5.02 ± 0.64 ms, *P* < 0.05), the time to 50% action potential repolarization (APD_50,_ from 42.17 ± 0.83 ms–28.90 ± 1.90 ms), and the APD_90_ (from 160.67 ± 5.85 ms–81.78 ± 5.45 ms, *P* < 0.05) after exposure for 30 min. As shown in Supplementary Figure S3A, the APD was significantly shortened in a time-dependent manner after exposure to ZnO NPs.

Exposure to ZnO NPs-100 at lower concentrations (10^−6^–10^−5^ g/ml) had no significant effects on both APA and APD. However, ZnO NPs-100 at 10^−4^ g/ml significantly reduced APA, and shortened APD_10_, APD_50_, and APD_90_ as early as in 5 min in a time-dependent manner. As shown in Supplementary Figure S3B, ZnO NPs-100 at 10^−4^ g/ml further decreased the APA (from 106.50 ± 3.73 mV–76.83 ± 3.40 mV, *P* < 0.0001), APD_10_ (from 11.18 ± 2.43 ms–5.61 ± 1.05 ms, *P* < 0.001), APD50 (from 28.92 ± 3.88 ms–13.55 ± 1.37 ms, *P* < 0.0001), and APD_90_ (from 98.07 ± 17.43 ms–41.12 ± 6.41 ms, *P* < 0.0001) after exposure for 30 min.

### 3.4 ZnO NPs did not affect the current densities of I_k1_

Based on the finding that the two types of ZnO NPs have no significant effect on the RP of cardiomyocytes, we propose that ZnO NPs may not affect the I_K1_ channel, which is known to be responsible for maintaining RP. To verify this hypothesis, we recorded I_K1_ currents in neonatal mouse cardiomyocytes with or without ZnO NPs exposure using the whole-cell patch-clamp technique. As shown in Supplementary Figure S4, neither ZnO NPs-40 (Supplementary Figures S4A,C) nor ZnO NPs-100 (Supplementary Figures S4B,D) significantly affected the current densities of I_K1_ channel, even at the highest concentration (10^−4^ g/ml).

### 3.5 ZnO NPs decreased the current densities and altered the kinetics of I_Na_

**Figure 3:**
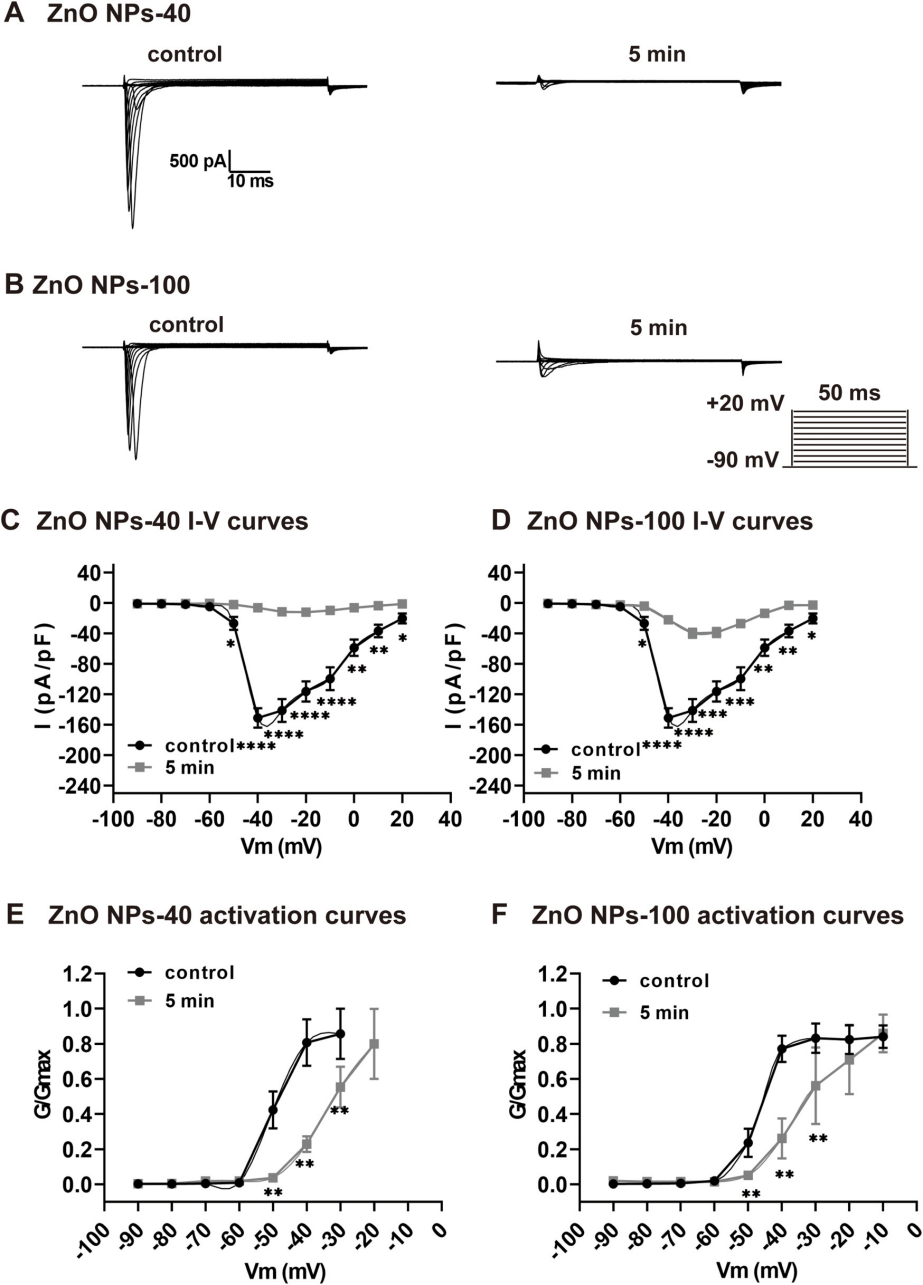
Rapid inhibitory effect of ZnO NPs on INa channel in NMVMs. **(A,B)** Typical I_Na_ currents recorded before and after exposure to ZnO NPs-40 and ZnO NPs-100 at 10^−4^ g/ml for 5 min, respectively. **(C,D)** Corresponding I–V curves of I_Na_ channels before and after exposure to ZnO NPs-40 and ZnO NPs-100. **(E,F)** The activation curves of I_Na_ channels at baseline and after exposure to ZnO NPs-40 and ZnO NPs-100 at 10^−4^ g/ml for 5 min. *P < 0.05, *P < 0.01, ***P < 0.001, ****P < 0.0001. *n* = 5.

We further recorded sodium channel currents (I_Na_) to investigate the mechanism that underlined the acute toxicological changes since both APA reduction and PR prolongation are correlated with I_Na_. As shown by ECG and AP results, exposure of ZnO NPs induced electrophysiological alteration as early as in 5 min. We found that both size of ZnO NPs dramatically decreased I_Na_ current (Figures 3A,B), and the current density that were calculated by normalized with membrane capacitance (Figures 3C,D) in 5 min. At −40 mV voltage stimulation, the I_Na_ current density (pA/pF) decreased from −155.59 ± 43.24 to −3.94 ± 0.69 (*P* < 0.01) after exposure to ZnO NPs-40 (10^−4^ g/ml), whereas decreased from −150.89 ± 12.65 to −6.13 ± 1.74 (*P* < 0.0001) after exposure to ZnO NPs-100 (10^−4^ g/ml) respectively compared to solvent control. In addition, we also calculate the kinetics of I_Na_ since it also plays an important role in the function of solidum channels and the shape of AP. As shown in Figures 3E,F, both ZnO NPs-40 and ZnO NPs-100 shifted the activation curve of the I_Na_ channels to the depolarization direction, indicating that ZnO NPs inhibited the activation of sodium channels. Surprisingly, the I_Na_ could not be detected after 5 min exposure, indicating the time-dependence of the toxic effect of ZnO NPs exposure.

### 3.6 ZnO NPs inhibit current densities of I_Ca−L_

**Figure 4:**
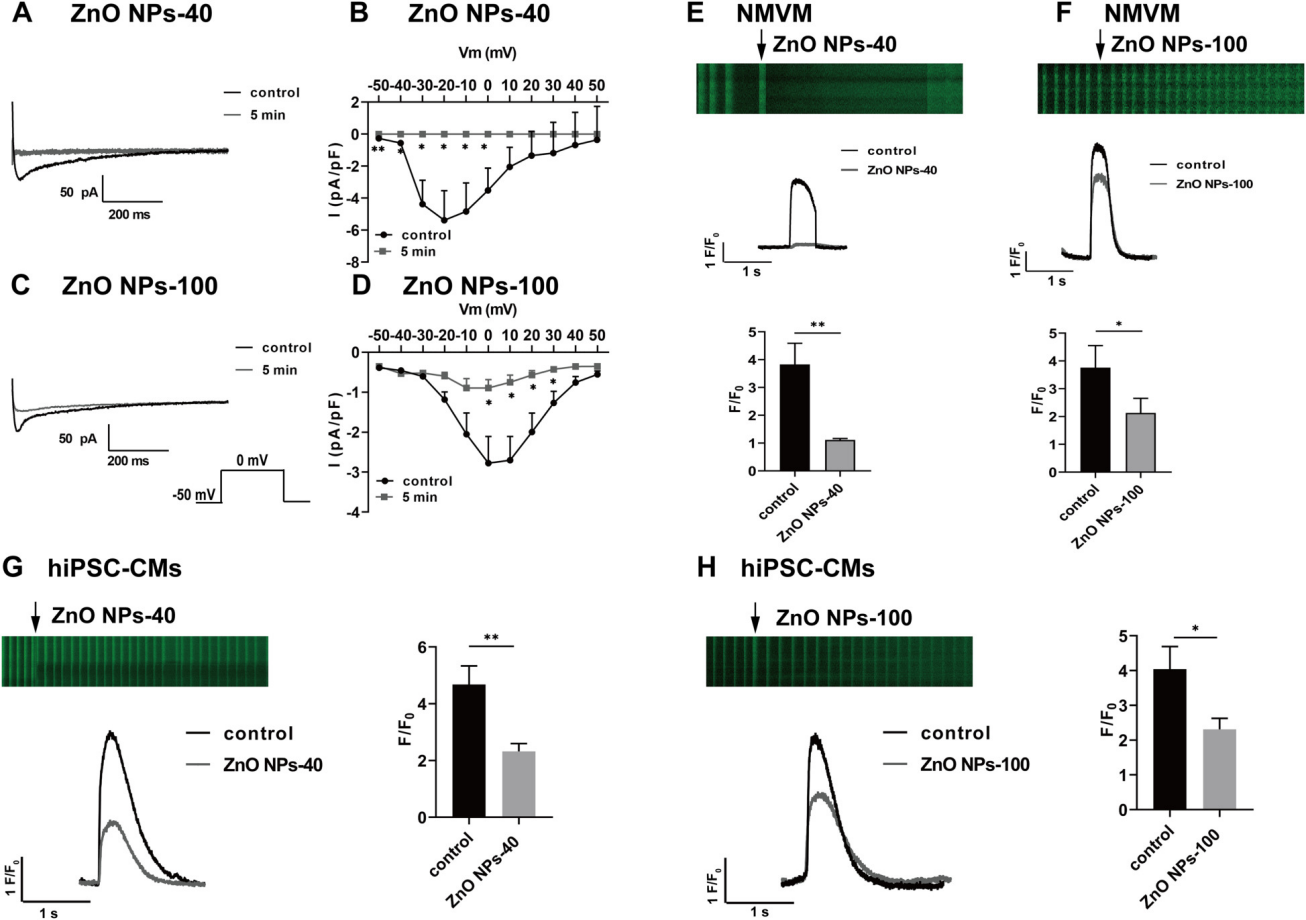
Zno NPs suppress I_Ca−L_ currents and calcium transients in NMVMs and hiPSC-CMs. **(A,C)** Typical I_Ca−L_ current traces before and after exposure to ZnO NPs-40 and ZnO NPs-100 for 5 min, respectively. **(B,D)** I-V curves of I_Ca−L_ channels before and after exposure to ZnO NPs-40 and ZnO NPs-100 for 5 min, *n* = 6. **(E,F)** Representative line scan image of cardiomyocytes loaded with the Ca^2+^ indicator fluo-4 AM (5 μM) and exposed to ZnO NPs-40 and ZnO NPs-100 at a concentration of 10^−4^ g/ml. **(G,H)** Representative line scan image of hiPSC-CMs loaded with the Ca^2+^ indicator fluo-4 AM (5 μM) and exposed to ZnO NPs-40 and ZnO NPs-100 at a concentration of 10^−4^ g/ml. ns. not significant, *P < 0.05, **P < 0.01, *n* = 10.

We next analyze the effects of ZnO NPs exposure on calcium channel current I_Ca−L_, which contributes to plateau phage of action potential and plays a pivotal role in APD. Both size of ZnO NPs dramatically decreased I_Ca_ current (Figure 4A,B), and the current density that were calculated by normalized with membrane capacitance (Figure 4C,D) in 5 min. Notably, ZnO NPs-40 exposure almost completely reduced I_Ca−L_ of the neonatal mouse ventricular myocytes in 5 min, indicating a size-dependent toxicity of ZnO NPs exposure.

### 3.7 ZnO NPs inhibit calcium transients in both NMVMs and hiPSC-CMs

The electrophysiological changes shown above, especially the alteration of I_Ca−L,_ could induce dynamic changes of intracellular Ca^2+^. We then recorded calcium transient from isolated mouse cardiomyocytes loaded with the Ca^2+^ indicator fluo-4 AM. The calcium transients were calculated by normalized fluorescence (F/F0), where F is the peak fluorescence intensity and F0 is the average fluorescence in the resting or diastolic state. As shown in Figures 4E,F, exposure to ZnO NPs-40 or ZnO NPs-100 at 10^−4^ g/ml for 5 min significantly reduced calcium transients, with greater reduction of ZnO NPs-40 (from 3.92 ± 0.87–1.10 ± 0.12) compared to ZnO NPs-100 (from 3.84 ± 0.91–2.21 ± 0.46). To further improve the translational significance of our finding, we also evaluate the acute effects of ZnO NPs exposure on human iPSC-CMs. As shown in Figures 4G,H, both ZnO NPs-40 and ZnO NPs-100 at 10^−4^ g/ml significantly reduced the amplitude of calcium transients in 5 min, with greater reduction of ZnO NPs-40 (from 4.32 ± 0.68–2.13 ± 0.19) compared to ZnO NPs-100 (from 4.05 ± 0.72–2.37 ± 0.23).

### 3.8 ZnO NPs inhibit cardiac function *in vivo*

**Figure 5:**
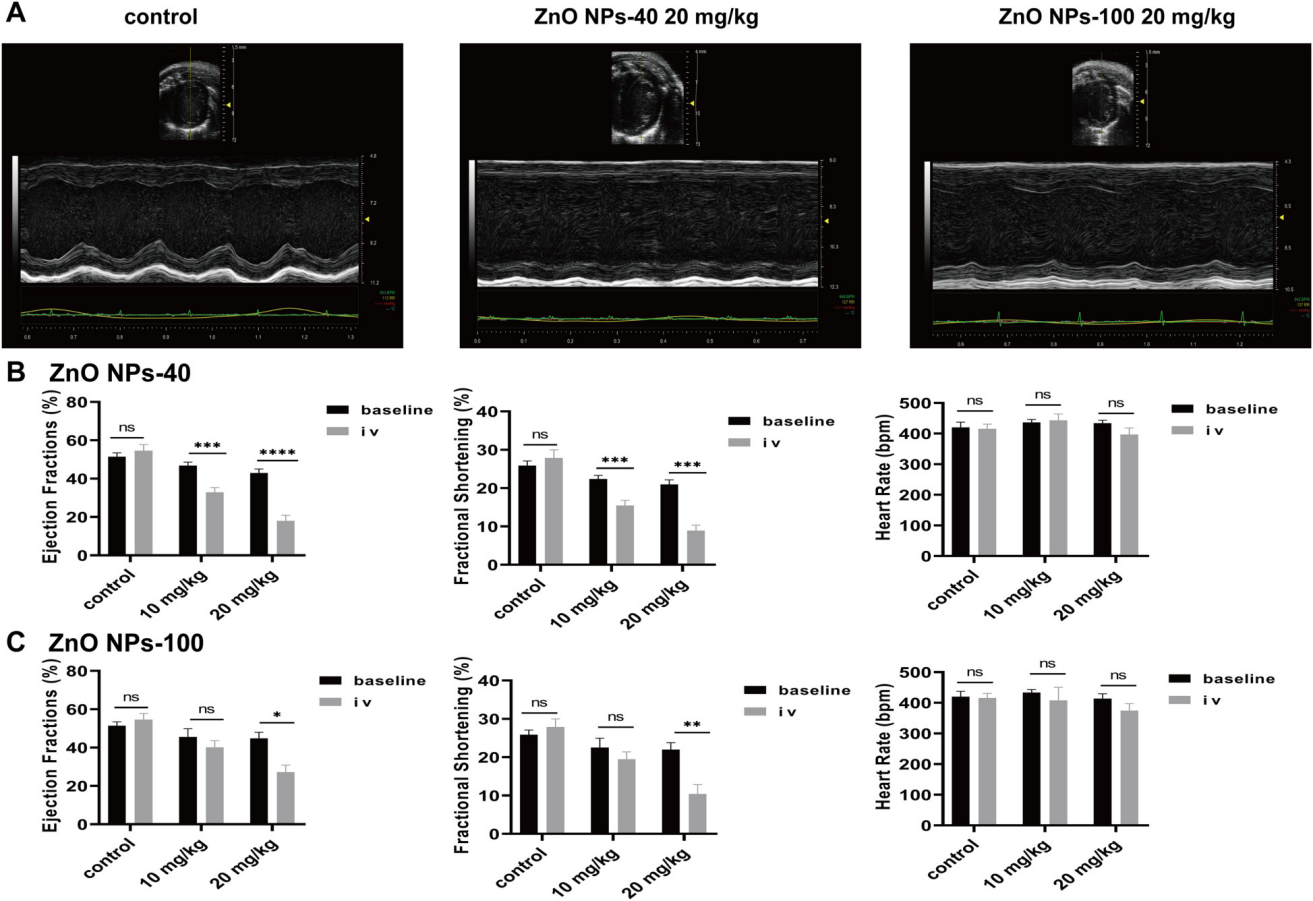
Zno NPs suppress cardiac contractility. **(A)** Representative echocardiographic images of mice after normal saline tail vein injection, 20 mg/kg ZnO NPs-40 and ZnO NPs-100 respectively. **(B)** Changes in left ventricular ejection fraction, short-axis shortening rate, and heart rate of mice after tail vein injection of normal saline and 10–20 mg/kg of ZnO NPs-40. **(C)** Changes in left ventricular ejection fraction, short-axis shortening rate and heart rate in mice after tail vein injection of normal saline and 10–20 mg/kg ZnO NPs-100. ns. not significant, *P < 0.05 vs baseline, **P < 0.01 vs. baseline, ***P < 0.001 vs. baseline, ****P < 0.0001 vs. baseline. *n* = 6.

The calcium transient of cardiomyocytes is one major factor that regulate contraction of heart. To further investigate the effects of ZnO NPs exposure on cardiac contractility, we carried out M-mode echocardiography of mice 5 min after ZnO NPs injection similar as for ECG recording. To avoid the disturbance of heart rates on contraction, we adjusted the depth of anesthesia of mice individually and dynamically so that the average heart rates reached around 400 beats per min. As shown in Figure 5A, both ZnO NPs-40 and ZnO NPs-100 had inhibitory effects on cardiac contraction. Exposure to 10 mg/kg ZnO NPs-40 decreased LVEF% from 46.84 ± 0.6–32.92 ± 0.82, and decreased LVFS% from 22.39 ± 0.27–15.50 ± 0.44. In addition, exposure to ZnO NPs-40 at 20 mg/kg decreased the LVEF% from 42.96 ± 0.77–18.02 ± 1.29, and decreased LVFS% from 20.96 ± 0.45–8.92 ± 0.71, indicating a dose-dependent effects (Figure 5B). In contrast, exposure to ZnO NPs-100 at 10 mg/kg had no significant changes in LVEF% and LVFS%, whereas exposure to ZnO NPs-100 at 10 mg/kg significantly decreased the LVEF% from 44.83 ± 3.12–27.15 ± 3.72 and LVFS% from 21.99 ± 1.78–10.42 ± 2.43, further indicating the size-dependent effects of ZnO NPs induced toxicity (Figure 5C).

### 3.9 The acute toxicity of ZnO NPs is independent of oxidative stress

**Figure 6:**
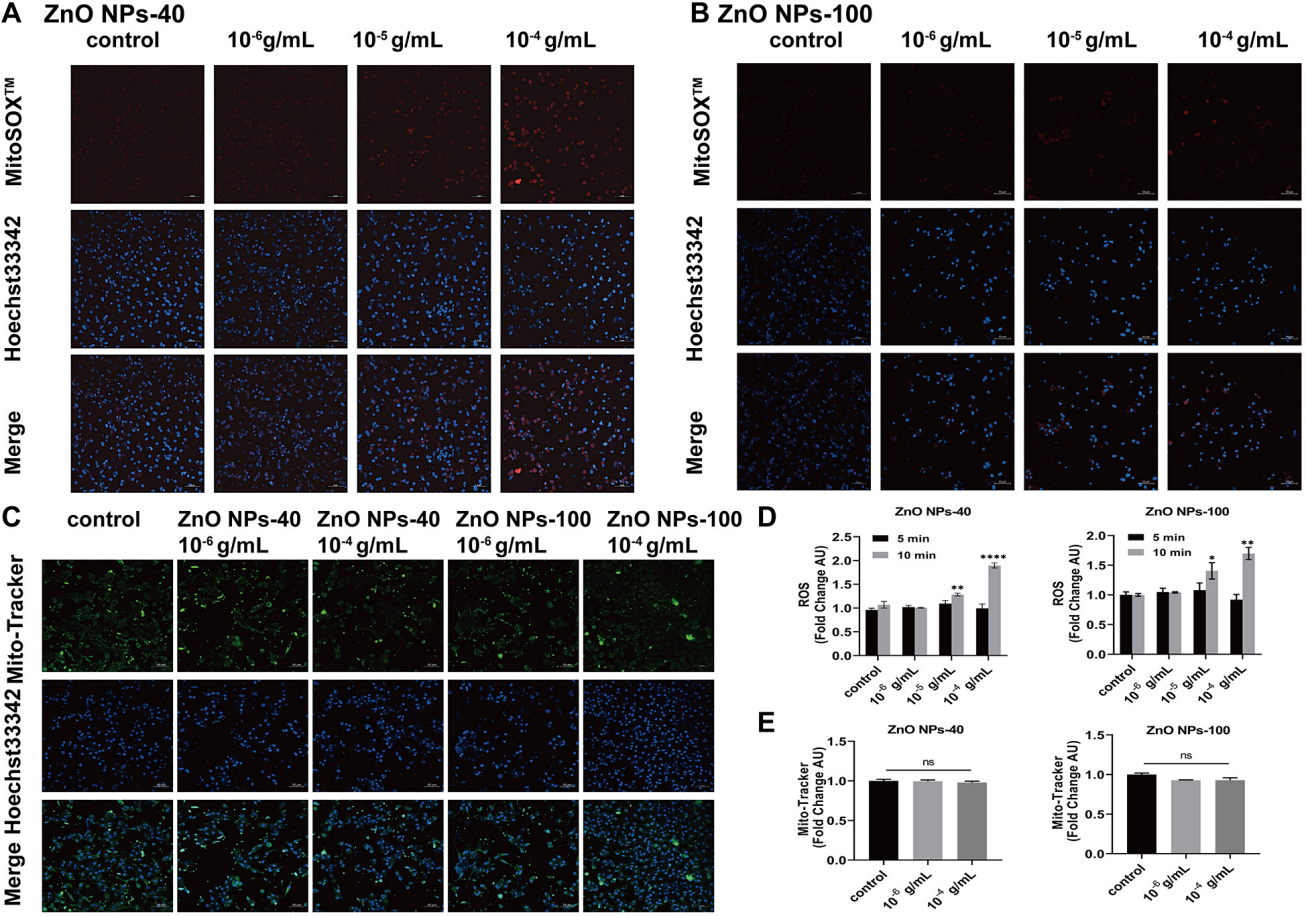
Effects of ZnO NPs on ROS production and mitochondrial density. **(A)** Fluorescence images of MitoSOXTM Red labeled ROS in NMVMs treated with 10^−6^ g/ml–10^−4^ g/ml ZnO NPs-40 for 10 min. **(B)** Fluorescence images of MitoSOXTM Red labeled ROS in the primary ventricular myocytes treated with 10^−6^ g/ml–10^−4^ g/ml ZnO NPs-100 for 10 min. **(C)** Fluorescence images of Mito-Tracker Green labeled mitochondrion in NMVMs treated with two kinds of ZnO NPs for 10 min, respectively. **(D)** Statistical results of average fluorescence intensity of ROS level in NMVMs exposed to two types of ZnO NPs for 5 min or 10 min respectively. **(E)** Statistical analysis of average fluorescence intensity of mitochondria in NMVMs exposed to two kinds of ZnO NPs for 10 min respectively. Scale bar, 50 μm. ns. no significance, *P < 0.05 vs. control, **P < 0.01 vs. control, ***P < 0.001 vs. control, ****P < 0.0001 vs. control. *n* = 3.

Previous studies have shown that ZnO NPs exit toxic effects via extensive ROS generation in mitochondria (28, 29). To verify whether ZnO NPs exposure affect electrophysiological changes via ROS, we further exposed cultured NMVMs to the two types of ZnO NPs at concentrations ranging from 10^−6^ g/ml–10^−4^ g/ml for either 5 min or 10 min. MitoSOX staining indicated that there were no significant changes of intracellular ROS levels compared to the control group after 5 min exposure (data not shown here). However, after 10 min of exposure, both types of ZnO NPs at higher concentration (10^−5^ g/ml and 10^−4^ g/ml) significantly elevated the levels of intracellular ROS, as illustrated in Figures 6A–D. We also examined the effects of ZnO NPs on mitochondrial function by Fluorescence images of Mito-Tracker Green. As shown in Figures 6C–E, both types of ZnO NPs at concentrations of 10^−6^ g/ml and 10^−4^ g/ml have no effects on the density of mitochondria in ventricular myocytes, even after 10 min exposure.

### 3.10 ZnO NPs did not cause cardiac inflammation or structural alterations

We further investigated whether ZnO NPs caused acute toxic effects via inflammation or structural changes in heart. H&E staining showed that acute exposure to ZnO NPs at a dose of 20 mg/kg, even for 10 min, did not cause any aggregation of inflammatory cells in the heart (Supplementary Figure S5). At cellular levels, TEM images (Supplementary Figures S6A–C) show that after 5 min exposure, none of the two types of ZnO NPs were endocytosed into the cells. Moreover, both ZnO NPs-40 and ZnO NPs-100 had no significant effects on LDH release even at 10^−4^ g/ml concentration (Supplementary Figures S6D,E), indicating the integrity of cell membrane of NMVMs after 5 min exposure.

## 4 Discussion

Although ZnO NPs pollution has raised high concern of their safety, their potential risk after acute exposure, especially for cardiovascular system, is still unknown. Here we selected two different sizes of ZnO NPs (NP-40 and NP-100) to investigated their effect on heart both *in vitro* and *in vivo*. Our results revealed that acute exposure of ZnO NPs is arrhythmogenic and prone to heart failure.

Previous reports have shown that ZnO NPs with particle sizes ≤100 nm are among the most commonly used in food, cosmetics, and biomedicine industries (30–35). For example, the average particle size of ZnO NPs in sunscreen products is typically 20–50 nm as indicated by ISO/TS 19007:2023. This particle size range has also been widely adopted in published toxicological studies of ZnO NPs. For instance, Li et al. utilized ZnO NPs with the particle size ≤50 nm to investigate their potential harmful effects on hiPSC-CMs (20). Milla C. et al. evaluated the toxicity of ZnO NPs, with around 100 nm size analyzed by TEM, on H9C2 rat cardiomyoblasts (16). Nagarajan et al. demonstrated that ZnO NPs with particle sizes <100 nm could induce cardiovascular toxicity (17). Poier et al. observed that continuous exposure to ZnO NPs (average size of 45–55 nm, concentrations exceeding 25 μg/ml) reduced cell viability of HUVECs, while induced DNA damage and hindered angiogenesis even at subcytotoxic levels (12). It has been revealed that the toxic effects of ZnO NPs are not only size-dependent, but also dose-dependent. *In vitro*, ZnO NPs with a concentration ranged from 1–100 μg/ml promoted atherosclerosis progression and accelerate foam cell formation in a dose-dependent manner (14). Chuang et al. also found that ZnO NPs at highest concentration of 150 μg/ml dramatically decrease the viability of human coronary artery endothelial cells (36). In animal studies, repeat oral administration of higher dose of ZnO NPs (208 mg/kg/day) promoted malignant colonic inflammation and malignant transformation compare to lower dose (26 mg/kg/day) or solvent control (37). In addition, continuous intraperitoneal injection of high dose of concentration of ZnO NPs (150 mg/kg ZnO NPs) induced ferroptosis of spermatocytes in mouse testes (38). Considering factors such as acute exposure scenarios, administration routes, previous findings as above, and our own preliminary data, we select two sizes of ZnO NPs (40 and 100 nm) with gradient doses in this study.

The various exposure routes of ZnO NPs, including oral, intraperitoneal, inhalational, intravenous, and percutaneous administration, have well been documented to understand toxicological effects and mechanism (39). Intravenous exposure to ZnO NPs typically happens in medical settings, particularly for investigating immunotherapy for tumors (40, 41). Following single intravenously injection, ZnO NPs were rapidly distributed to different organs (42). Intravenous exposure of pregnant mice demonstrated that ZnO-NPs can cross the underdeveloped blood-brain barrier into fetal brain tissue and be internalized by microglia (43). After multiple tail vein injections, Zhu et al. noted the accumulation of ZnO NPs in mammary tissue that disrupted the epithelial barrier (44). In addition, Choi et al. reported that single intravenous injection of ZnO NPs (30 mg/kg) showed high risk of acute injuries compared to oral exposure at same dose (45). In this study, we administered ZnO NPs (from 5 mg/kg–30 mg/kg) via single intravenous injection to simulate acute exposure and evaluate its direct effects on the cardiovascular system, thus providing new perspective of ZnO NPs induced toxicology.

We demonstrated that both sizes of ZnO NPs could induce prolongation of the PR-interval, PVC, AVB, bradycardia, and asystole as indicated by ECG as early as 5 min post tail vein injection, which eventually led to animal death. The cardiotoxicity of ZnO NPs-40 in mice is higher than that of ZnO NPs-100. At the same dose, ZnO NPs-40 causes a more serious arrhythmia and higher mortality rate. The acute cardiac toxicity is similar to that of SiNPs, which induced bradycardia and sudden cardiac death in mice in a dose- and time-dependent manner (26). We also observed that some mice exhibited temporarily increased heart rate following ZnO NPs injection. This phenomenon may be attributed to the pain or discomfort response associated with the injection procedure that causes a short-term release of catecholamines.

We tried to explored the mechanisms underlying the electrophysiological alteration upon acute ZnO NPs exposure. TMP recording revealed that ZnO NPs have no effects on resting membrane potential, but significantly reduced action potential duration (APD) and action potential amplitude (APA). Since activities of various ion channel in the membrane contribute to the dynamics of TMP, we then record ion channel currents to correlate the changes in TMP. We found that both sizes of ZnO NPs have no effects on the current densities of I_K1_ channels, which account for the dominant ion channel to maintain RP (46). This effects of ZnO NPs exposure on RP were similar to that of SiNPs (26), but it is different from that of AgNPs and PtNPs, both of which significantly elevated the RP of cardiomyocytes (47, 48). We next record sodium channel currents I_Na_ because rapid depolarization of AP in ventricular myocytes is mainly caused by sodium current (I_Na_). As shown in the results, ZnO NPs rapidly inhibit both the current densities and voltage dependent of action of I_Na_ in a size-dependent manner, thereby reducing the APA of cardiomyocytes that serves as one main factor to affect cardiac conductance. Lastly we record L-type calcium channel current (I_Ca−L_) because it was responsible for not only repolarization and shape of AP of ventricular cardiomyocytes, but also the action potential upstroke that determine the APA of atrial ventricular node cell (49). Similarly, ZnO NPs rapidly inhibit the current densities of I_Ca−L_ in a size-dependent manner. We did not evaluate the effects of ZnO NPs on other channels involved in repolarization of AP, such as IKr and IKs, which are rapidly downregulated after birth and difficult to be detected in mouse cardiomyocytes (50). Therefore, the inhibitory effects of ZnO NPs on I_Na_ and I_Ca−L_ currents might together disturb cardiac conductance, and cause prolongation of PR-interval, AVB and even asystole as we observed.

One question may emerge regarding how ZnO NPs regulated I_Na_ and I_Ca−L_ channels. Currently there is no report to show the acute toxicity of ZnO NPs on cardiac electrophysiology. ZnO NPs could directly contact and interact with the channels, or regulate channel function indirectly. One possible mechanism might be attributed to oxidative stress and membrane injury induced by ZnO NPs exposure (51). In this study, ZnO NPs did not induced the release of LDH or ROS in NMVMs within 5 min, whereas the electrophysiological effects developed rapidly within 5 min both *in vivo* and *in vitro*, indicating a temporal dissociation between the acute impact and oxidative stress or membrane injuries. Furthermore, TEM analysis demonstrated that there was no endocytosis of ZnO NPs within 5 min exposure. H&E staining showed that the ZnO NPs did not trigger inflammation or cause cardiac structural changes. In addition, translation changes of ion channel genes, that might be regulated upon ZnO NPs exposure, could not contribute to the changes of ion channel currents, as membrane protein synthesis or degradation need more than 5 min. Therefore, we suggest that the acute toxic effects of ZnO NPs on cardiac electrophysiology were attributed to its direct interaction with the extracellular parts of ion channels to inhibit channel currents. Our inference is in line with, previous reported that ZnO NPs could alter the kinetics of Kv 11.1 via directly interaction with the outer surface of channels that were transfected into HEK293 cells (52).

In parallel with decreased I_Ca−L,_ we found ZnO NPs exposure rapidly reduce cardiac contractility of mouse heart in a dose-dependent manner as indicated by LVEF of echocardiography. Similarly, smaller size of ZnO NPs-40 caused more serious decrease in cardiac function. We then analyzed the calcium transient changes, the amplitude of which is regulated by I_Ca−L_ and highly linked to cardiac contractile function (53). As expected, we found ZnO NPs exposure quickly reduced the amplitude of calcium transient NMVM in 5 min. In recent years, human iPSC derived cardiomyocytes (hiPSC-CM) have been extensively utilized to predict chemical-induced cardiotoxicity (54, 55). In this study, we also observed acute inhibitory effects of ZnO NPs on calcium transients of hiPSC-CMs. indicating the potential toxicity of ZnO NPs exposure on human being.

There are still some limitations that need to be addressed in the future. First, although we have systematically investigated the acute effects of ZnO NPs exposure on both cardiac electrophysiology and contractility for the first time, the chronic effects of ZnO NPs exposure were not explored. Since the current doses (10 mg/kg for ZnO NPs-40, 30 mg/kg for ZnO NPs-100) are lethal, small doses should be utilized to provide further insight of different properties between acute and long-term cardiovascular risks. Second, while the predominant channel subtypes that account for I_Na_ and I_Ca−L_ are NaV1.5 and CaV1.2 respectively, other ion channels may also be involved the toxic effects. For example, NaV1.8 sodium channel, which belongs to the TTX-resistant sodium channel family, is expressed in Purkinje fibers and myocardium (56). Sotoodehnia et al. reported that the blocking NaV1.8 by its blocker A-803467 could cause a prolongation of the PR-interval in awake mice (57). The involvement of specific channel subtypes in ZnO NPs-induced impairment could be further delineated through pharmacological or molecular studies. Last, cardioprotective agents could be explored to facilitate the development of potential therapeutic interventions, to mitigate the cardiocytoxicity in clinical practice.

## 5 Conclusion

ZnO NPs exert acute and profound toxic effects on cardiac electrophysiology and contractile function. These acute effects are not associated with ROS generation or membrane injuries upon ZnO NPs exposure, but mediated by direct inhibition of I_Na_ and I_Ca−L_ channels, which account for reduced cardiac conductance and calcium transient amplitude. To our knowledge, this study is the first report that explore the cardiotoxicity of ZnO NPs in both mouse and human cardiomyocytes, providing more translational significance. Therefore, more attention should be paid to the utilization of ZnO NPs in the field of nanomedicine.

## Data Availability

The raw data supporting the conclusions of this article will be made available by the authors, without undue reservation.
