# Supplementary material for: ZnO nanoparticles induce acute arrhythmia and heart failure in mice by disturbing cardiac ion channels
Source: Front Cardiovasc Med. 2025 May 30;12:1569265. doi: 10.3389/fcvm.2025.1569265 (PMC12162645; doi:10.3389/fcvm.2025.1569265)
Supplement: Supplementary file 1 [file Supplementaryfile1.pdf]

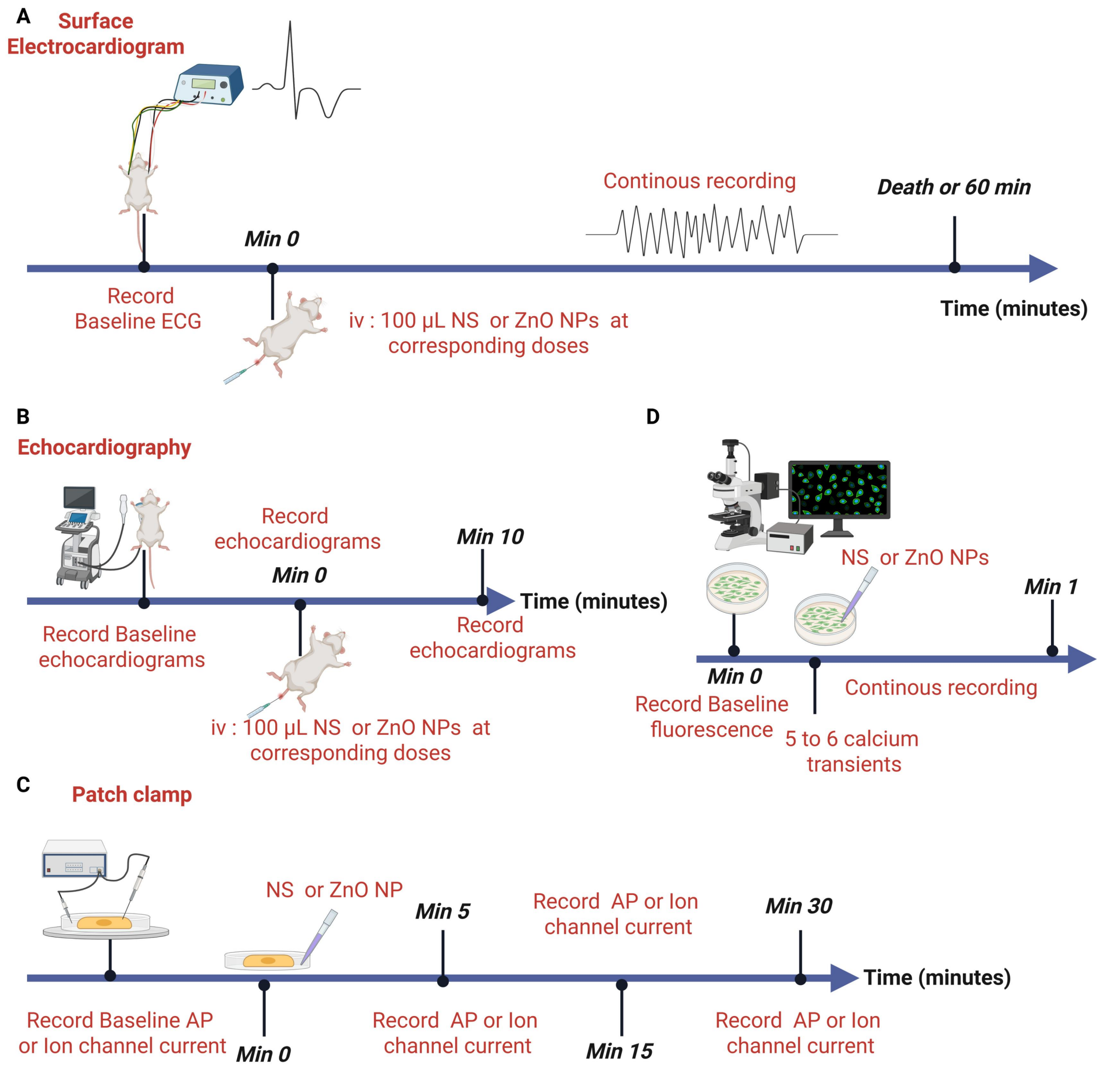

Fig.S1 The schematic diagram of the experimental models. A : Schematic diagram of ECG acquisition in the mouse model. B: Schematic illustration of echocardiogram acquisition in the mouse model. C : Schematic representation of action potentials or ion channel currents recorded from neonatal rat ventricular myocytes or hiPSC-CMs. D :Schematic representation of calcium transients recorded from neonatal rat ventricular myocytes or hiPSC-CMs. NS: Normal Saline.

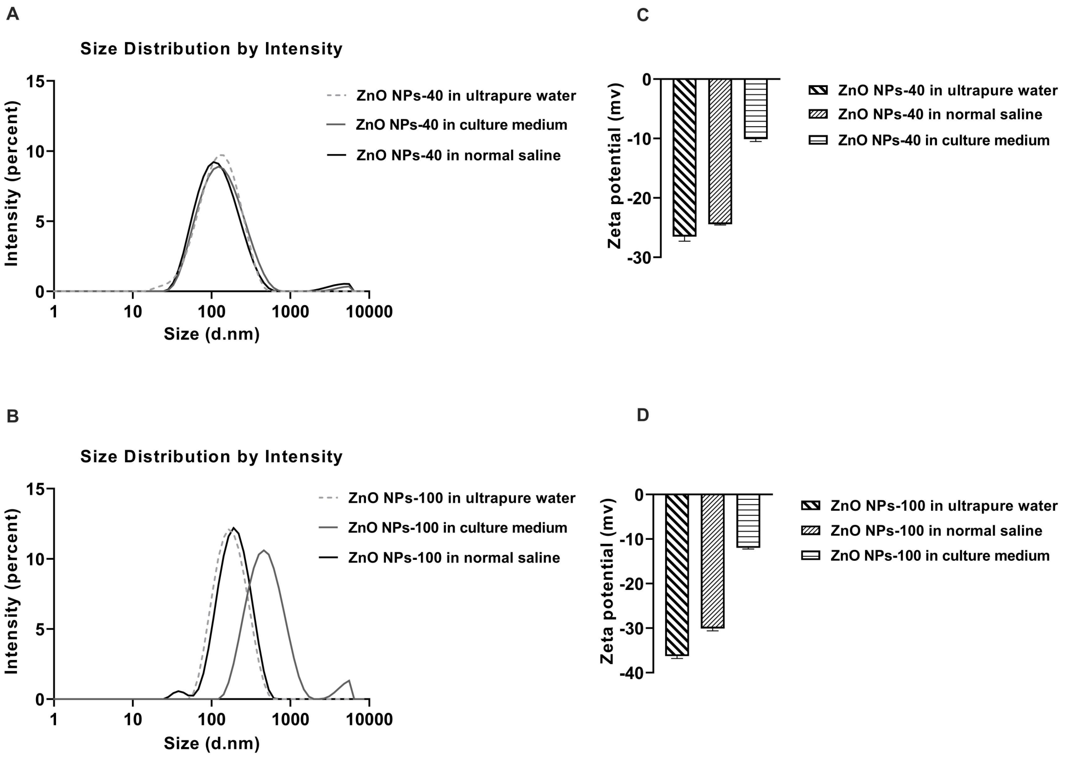

Fig.S2 The characterization of ZnO NPs. (A and B): Hydrodynamic diameters of ZnO NPs-40 and ZnO NPs-100 in different dispersion systems, respectively. (C and D): Zeta potentials of ZnO NPs-40 and ZnO NPs-100 in different dispersion systems, respectively. Abbreviations: ZnO NPs, zinc oxide nanoparticles; ZnO NPs-40, zinc oxide nanoparticles with diameters of 40 nanometers. ZnO NPs-100, zinc oxide nanoparticles with diameters of 100 nanometers.Values are presented as mean ± SEM. n = 3 per group, 3 separate experiments.

Table S1 Physicochemical Characterization of ZnO NPs

Dispersants		ultrapure water	normal saline	culture medium
Z-Average (nm)	ZnO NPs-40	105.37 ± 0.27	102.69 ± 0.17	111.96 ± 0.52
	ZnO NPs-100	151.60 ± 1.60	172.00 ± 2.49	441.40 ±1.68
Zeta Potential (mV)	ZnO NPs-40	-26.40 ± 2.05	-25.40 ± 0.37	-10.10 ± 0.39
	ZnO NPs-100	-36.30 ± 0.54	-30.10 ± 0.52	-12.40 ± 0.18
PDI	ZnO NPs-40	0.26 ± 0.00	0.27 ± 0.00	0.26 ± 0.00
	ZnO NPs-100	0.15 ± 0.01	0.20 ± 0.01	0.26 ± 0.02

Notes: Z-average, mean particle size measured by dynamic laser scattering technology

Abbreviations: PDI, polydispersity index. Values are presented as mean ± SEM.

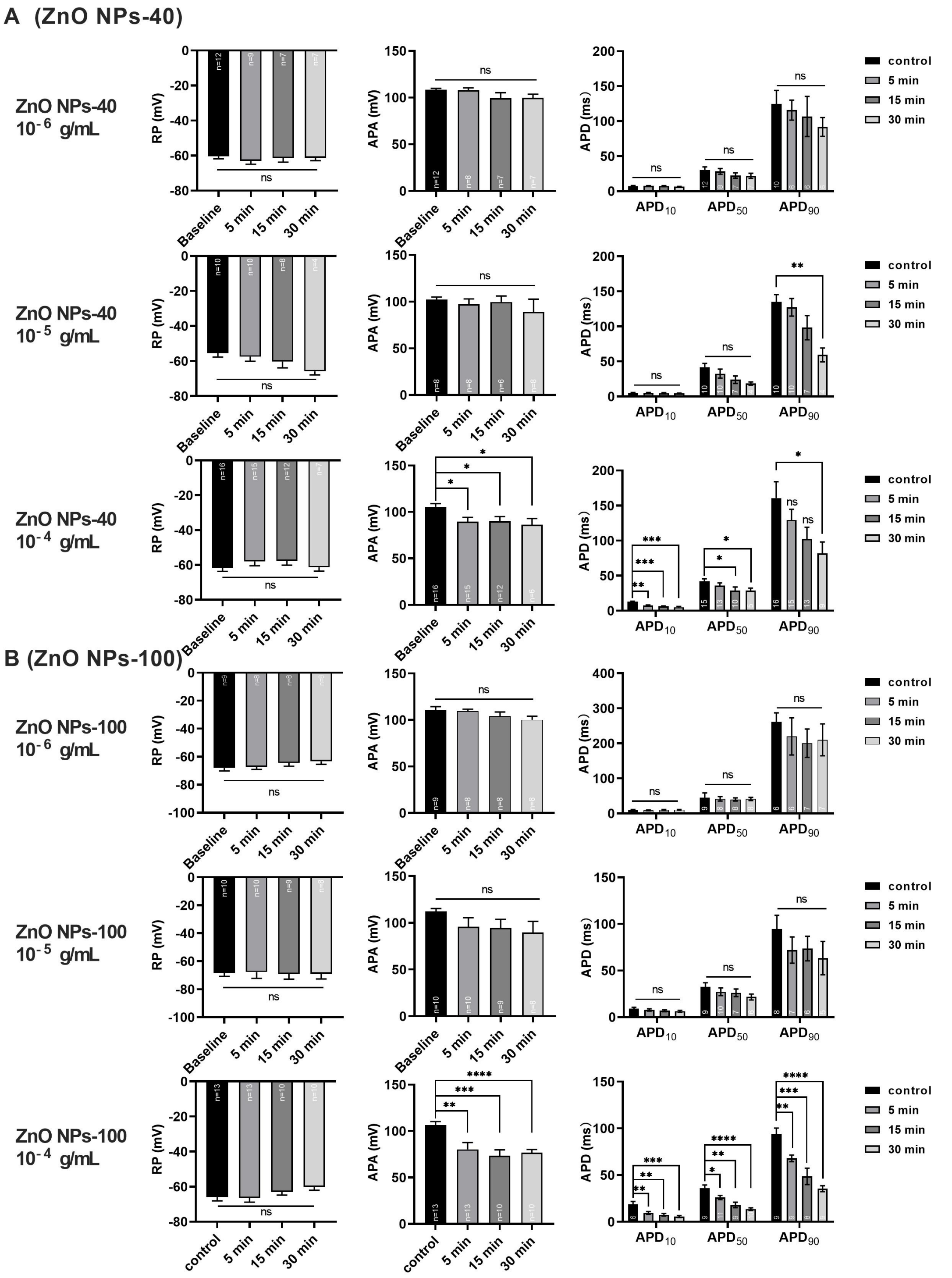

Fig.S3 Statistical results of parameters related to the effect of ZnO NPs on the transmembrane potential of neonatal mouse ventricular myocytes. A: Effects of ZnO NPs-40 (10^{-6}-10^{-4} g/mL) on RP, APA, and APD after 5-30 min exposure. B: Effects of ZnO NPs-100 (10^{-6}-10^{-4} g/mL) on RP, APA, and APD after 5-30 min exposure. ns. not significant, *P

< 0.05, ** $P < 0.01$, *** $P < 0.001$, **** $P < 0.0001$. Values are presented as mean ± SEM.

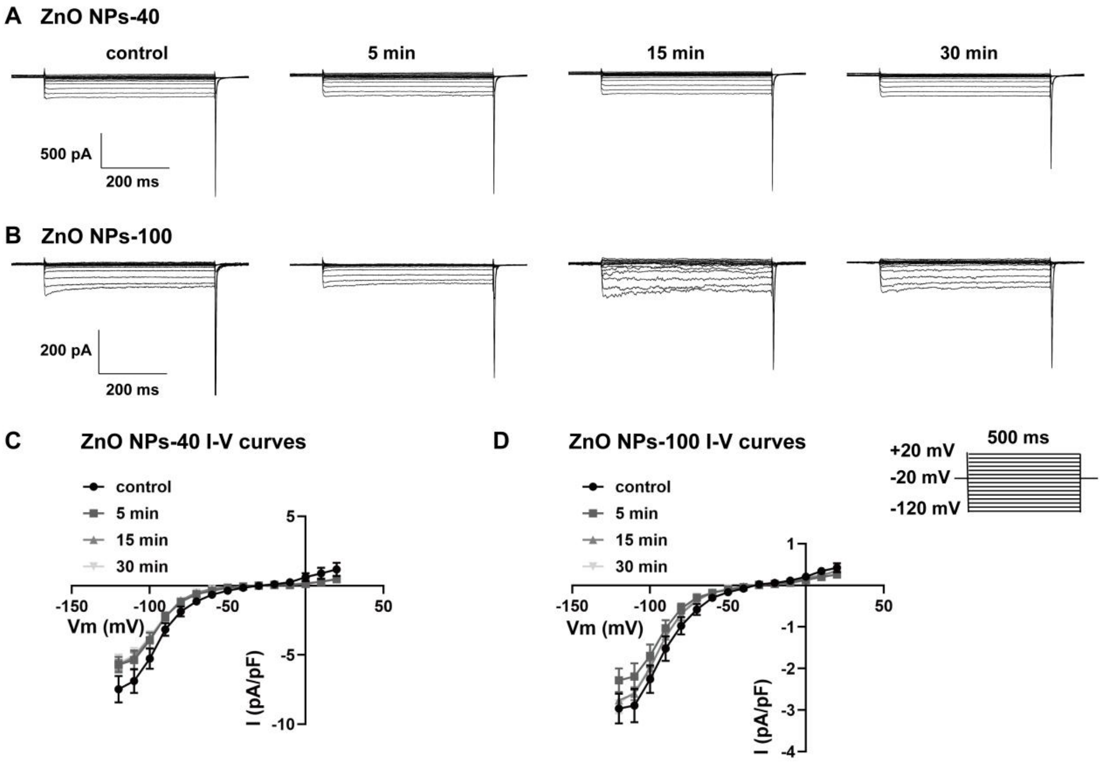

Fig.S4 ZnO NPs-40 and ZnO NPs-100 at a concentration of 10^{-4} g/mL had no significant effect on I_{K1} channel in neonatal mouse ventricular myocytes. A: Typical I_{K1} current trajectory diagram before and after exposure to 10^{-4} g/mL ZnO NPs-40 for 5-30 min. B: Typical I_{K1} current trajectory diagram before and after exposure to 10^{-4} g/mL ZnO NPs-100 for 5-30 min. C: I-V curves of I_{K1} channel, ZnO NPs-40 did not significantly change the current density of I_{K1}. D: I-V curves of I_{K1} channel, ZnO NPs-100 did not significantly change the current density of I_{K1} after 5-30 min of exposure.Values are presented as mean ± SEM. n = 3 cells for each ZnO NPs concentration.

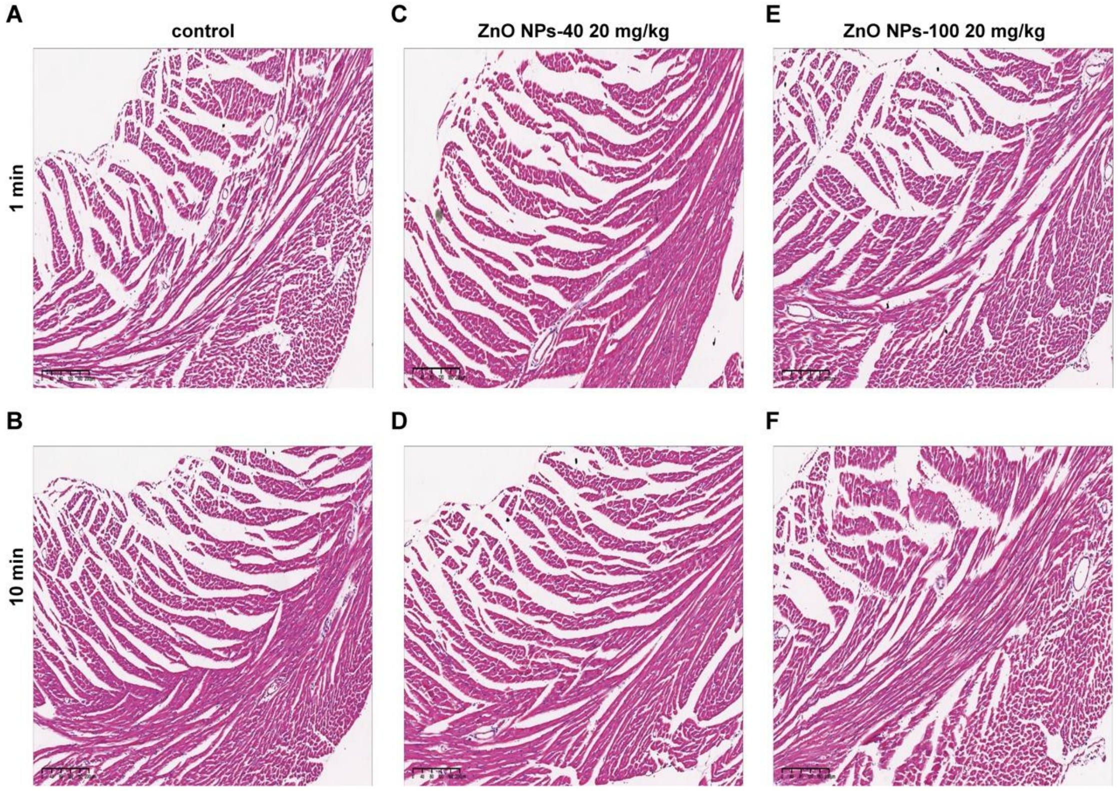

Fig.S5 Representative images of H&E staining of heart tissue. A and B: Injection with normal saline, exposure for 1 min and 10 min, respectively. C and D: Injection with ZnO NPs-40 at 20 mg/kg, exposure for 1 min and 10 min, respectively. E and F: Injection with ZnO NPs-100 at 20 mg/kg, exposure for 1 min and 10 min, respectively. n = 3 mice per group.

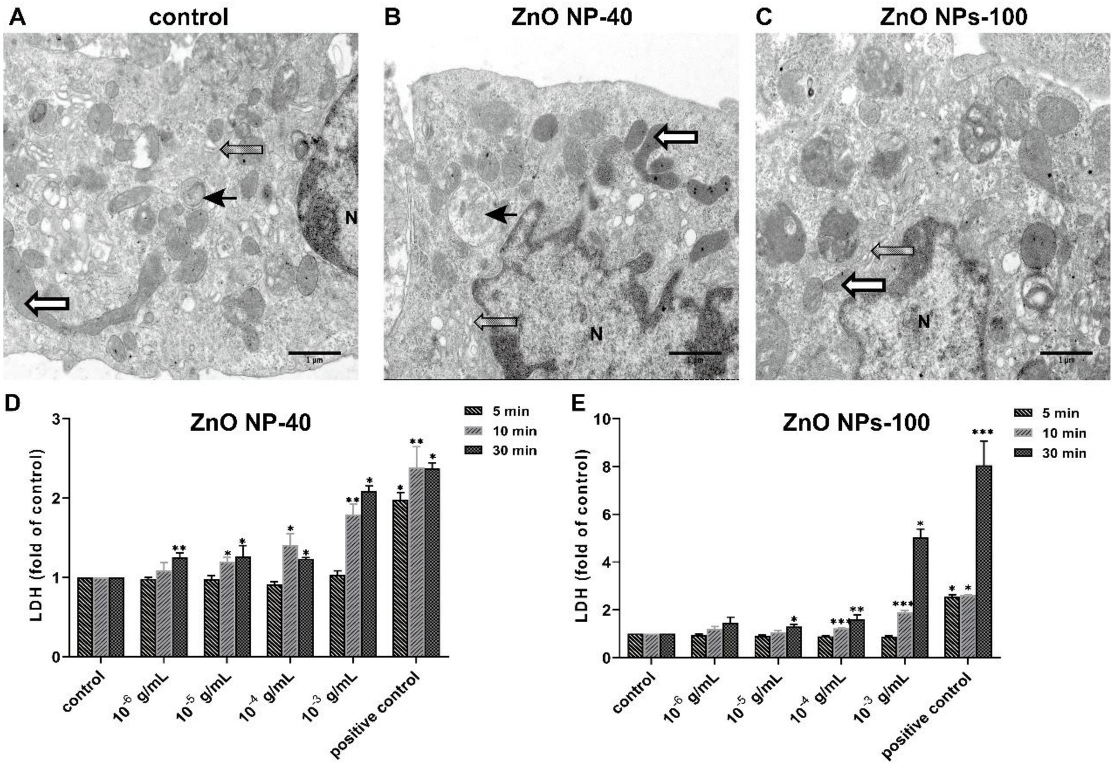

Fig.S6 The acute cardiotoxicity of ZnO NPs is not dependent on endocytosis into cells, nor does it damage cell membranes. A: TEM images of cells in the control group without nanoparticle treatment. B: TEM images of cells exposed to ZnO NPs-40 for 5 min. C: TEM images of cells exposed to ZnO NPs-100 for 5 min. D: Exposure to ZnO NPs-40 for 10 min increases the release of LDH at a concentration of 10^{-5} g/mL, and exposure to ZnO NPs-40 for 30 min increases the release of LDH at a concentration of 10^{-6} g/mL. E: Exposure to ZnO NPs-100 for 10 min increases the release of LDH at a concentration of 10^{-4} g/mL, and exposure to ZnO NPs-100 for 30 min increases the release of LDH at a concentration of 10^{-5} g/mL. Nucleus (N), Mitochondria (M, indicated by the wide black bar with white arrows), Sarcoplasmic reticulum (SR, indicated by the thin black arrow), Autophagic lysosomes (ASS, indicated by the black arrow). *$P < 0.05$ vs control, **$P < 0.01$ vs control, ***$P < 0.001$ vs control, ****$P < 0.0001$ vs control. Data from 3 separate experiments with n = 3 per group.Values are presented as mean ± SEM.
